# DiGeorge syndrome: Relevance of psychiatric symptoms in undiagnosed adult patients

**DOI:** 10.1007/s00508-018-1335-y

**Published:** 2018-04-18

**Authors:** Christoph Kraus, Thomas Vanicek, Ana Weidenauer, Tav Khanaqa, Mara Stamenkovic, Rupert Lanzenberger, Matthäus Willeit, Siegfried Kasper

**Affiliations:** 1 Department of Psychiatry and Psychotherapy, Clinical Department of General Psychiatry, Medical University of Vienna, Waehringer Guertel 18–20, 1090 Vienna, Austria; 2 Department of Psychiatry and Psychotherapy, Philipps-University Marburg, Marburg, Germany

**Keywords:** 22q11 deletion syndrome, Panic disorder, Depression, Psychiatric genetics, Anxiety disorders

## Abstract

DiGeorge syndrome or 22q11.2 deletion syndrome is one of the most common genetic microdeletion syndromes in humans. In addition to physical manifestations, DiGeorge syndrome is associated with a high prevalence of psychiatric disorders, such as intellectual disability, schizophrenia and attention-deficit/hyperactivity disorder. Usually, the diagnosis of DiGeorge syndrome is made in early childhood. This article reports on the late diagnosis of a patient with panic disorder and comorbid major depression at the age of 51. Since genetic testing was not available before the 1990s, there might be many over 40-year-old patients, who remained undiagnosed. Psychiatric symptoms exhibit distinctive developmental trajectories and many of these exhibit an increase in incidence during adulthood. Hence, undiagnosed adult DiGeorge patients might present in psychiatric services. As in this case, a correct diagnosis of DiGeorge syndrome in adults may help to improve treatment and outcome.

## Introduction

The 22q11 deletion syndrome (DS), also known as DiGeorge or velocardiofacial syndrome, is one of the most common microdeletion syndromes in humans. It occurs in 1 in every 3000–6000 births and is equally distributed between males and females [1, 2]. The median age at diagnosis in children with congenital heart disease in a Swedish sample from 1991–2000 was 3.9 years [1]. Since lifetime support and early management of disease complications enhance quality of life in DiGeorge syndrome patients [3], early diagnosis is crucial. Genetic tests were not available until the mid-1990ies in Austria, therefore a substantial number of patients might be undiagnosed. Based on population data (Statistics Austria, www.statistik.at) and incidence rates, approximately 1800 patients with 22q11 DS aged older than 18 years could exist, for whose sake a correct diagnosis would be an important step towards targeted treatment.

In infants, DiGeorge syndrome typically presents with the triad immunodeficiency (~75% of patients), congenital cardiac anomalies (~75%) and hypocalcaemia due to hypoparathyroidism (~50%). However, a large number of further symptoms are known, which comprise palatal abnormalities (~75%), gastrointestinal problems such as reflux disease or dysmotility (~30%), genitourinary anomalies such as renal agenesis (~30%), hernia (~1%), neuronal tube defects, polydactyly, anomalies in the spine, face, ear, nose, eyelids or eyes (e.g. hypertelorism), malignancies or seizures, early-onset Parkinson disease (~30%) and psychiatric disorders (~60%) (see [4] for detailed pathophysiological mechanisms and comprehensive review). Variability in phenotype and severity is high, the clinical presentation is heterogeneous and therefore, the diagnosis in non-typically presenting patients is easily overlooked.

DiGeorge syndrome constitutes an increased risk for psychiatric diseases, such as intellectual disability, schizophrenia, attention deficit hyperactivity disorder or anxiety disorders (Table 1). Given the heterogeneity of symptoms and the unavailability of genetic testing in primary care settings or smaller community hospitals, patients might remain undiagnosed. Patients, who lack easily recognizable manifestations, such as cardiac defects, immunodeficiency and facial malformations might grow old without the need for substantial medical interventions. But undiagnosed patients exhibit an increased likelihood for psychiatric symptoms with progressing age. This is why the medical community must be informed of the case reported here to consider this genetic syndrome in clinical practice.

**Table 1:** Cumulative incidence (in %) for psychiatric diseases on 22q11.2 deletions and controls

Disease	ICD-10	Controls	Cases	Cases/controls
Any psychiatric disorder	F00–F99	10.05 (9.40–10.74)	30.32 (22.69–40.51)	3.0
Schizophrenia and related disorders	F20–F29	1.37 (1.12–1.67)	7.23 (3.04–17.16)	5.3
Mood disorders	F30–F39	3.14 (2.77–3.57)	5.87 (2.17–15.88)	1.9
Neurotic, stress-related, and somatoform disorders	F40–F48	4.68 (4.24–5.15)	7.03 (3.09–15.99)	1.5
Intellectual disability	F70–F79	0.73 (0.57–0.93)	15.08 (9.90–22.98)	20.7
Pervasive developmental disorders	F84	1.40 (1.19–1.64)	11.65 (7.08–19.18)	8.3
Childhood autism	F84.0	0.40 (0.29–0.53)	3.40 (1.40–8.26)	8.5
Behavioral and emotional disorders with onset usually occurring in childhood and adolescence	F90–F98	3.43 (3.10–3.80)	8.69 (4.76–15.87)	2.5

Figures represent percentages and are taken from [5]

Numbers in brackets represent 95% confidence intervals

*ICD-10* international classification of diseases and related health problems, 10^th^ revision

The pathophysiology of DiGeorge syndrome consists of microdeletions of 1.5–3 million base pairs on the long arm of chromosome 22 [2], which is one of the most structurally complex regions in the genome [4]. There are approximately 90 genes in a typical 3Mb 22q11.2 locus, which are hemizygously deleted. Several mouse models or single gene knockouts provide good translational evidence for 22q11 deletion syndrome [2]. Once diagnosis is suspected, genetic testing is necessary to determine the molecular pathophysiology of DiGeorge syndrome. Fluorescence in situ hybridization (FISH) with specific probes is able to probe the critical region of chromosome 22. Nowadays, FISH is increasingly being replaced by more modern tests, such as microarrays, SNP arrays or multiplex PCR methods.

## Case presentation

### Psychiatric history

A 51-year-old man was admitted to our university hospital in September 2016 with panic attacks, dyspnea and chronic obstructive pulmonary disease (COPD) grade IV. During panic attacks, the patient experienced a feeling of heart-race (but heart frequency remained below 100/min), cramps in the upper extremities and fear of death. In addition, the patient suffered from chronic abdominal pain (over all quadrants). Therefore, he had previously been admitted many times to hospitals near his hometown at medical and surgical departments. Since no organic causes for the symptoms were identified, the patient was referred to a local psychiatric department where he was admitted as an inpatient seven times between 2014 and 2016. The patient increasingly developed subtle memory and concentration deficits, depressed mood, and ruminations, predominantly about his deteriorating health. Because he relinquished hopes of recovery from abdominal pain, for the first time in his life in 2016, he made a suicide attempt by taking an unknown amount of trazodone and triazolam tablets. He was discharged from the psychiatric ward of the community hospital after stabilization of acute suicidal thoughts 1 week before being admitted to our hospital.

In the initial psychiatric examination, perception, memory and concentration were reduced. The thought flow was reduced, he exhibited depressed mood and ruminations, the patient felt sick, hopeless, and had increased feelings of guilt (mainly for seeking help from his family during panic attacks). His affect was reduced, there was inner tension with concomitant low levels of energy and increased motor activity. His mood deteriorated during the day with ruminating thoughts during the evenings and nights. Consequently, he had difficulties falling asleep, sleeping through the night, decreased total sleep time, early morning awakening and fatigue during the day. Occasionally, he exhibited anxiety, with a feeling of rapid heartbeat, panic and fear of death. These panic attacks were sometimes associated with dyspnea. The psychiatric examination was otherwise normal. In particular, there was no suicidal ideation or suicidality. Hence, a diagnosis of panic disorder with comorbid major depression (MD) with a current severe episode was made.

The patient was unemployed, had previously worked as a waiter and lived alone in a small town (<5000 inhabitants). He had no current partner or children. The patient was supported by the department’s social worker. He maintained social contact with his sister and two brothers, all of whom were psychiatrically healthy and highly functioning. The patient was described as a life-time worry-child by his sister. She also described a single short psychotic episode in 2013 with delusions of grandiosity.

Neurocognitive testing (carried out after improvement of the severe depressive episode) showed crystallized intelligence at an average, fluid intelligence at low average level (Multiple Choice Vocabulary Intelligence Test, MWT-B: 94; Basic Intelligence Functions, IBF, IQ: 82), there were no clinical signs of rapid onset dementia. An electroencephalogram (EEG) exhibited scattered theta waves over all leads, cranial magnetic resonance imaging (MRI) was not tolerated by the patient due to claustrophobic reactions.

On admission, the daily psychopharmacological therapy consisted of olanzapine 10 mg and trazodone 150 mg. At our hospital, sertraline was established as an antidepressant and anti-anxiety medication and dosed up to 250 mg. Serum concentration of sertraline was 50 ng/ml at 250 mg p.o.q.d. (reference value: 10–150 ng/ml at 250 mg). Due to better augmentative and side-effect profiles, olanzapine was changed to amisulpride and left at 50 mg at night. As additional anti-anxiety therapy, pregabaline was slowly titrated to 300 mg, thereby taking advantage of low potential of respiratory depression. With this combinatory antidepressant and anti-anxiety treatment, the patient achieved marked improvement of mood within 4 weeks. Anxiety and inner tension levels dropped, there were no further panic attacks during the stay in hospital.

Additionally, the patient was treated in psychotherapeutic groups at our ward but participation in physiotherapy and occupational therapy was poor. His favorite pastime at our ward was playing cards on his laptop. A successful relocation to a nursing home in Vienna was initiated by the social worker team.

### Medical history

In the initial medical examination, the patient weighed 80 kg at 179 cm, had mild edema in both legs, noisy breathing (mild wheezing) and reported diffuse but severe abdominal pain that had started 6 months ago. Previously performed examinations (gastroscopy, coloscopy, X‑ray) did not find a somatic cause for the pain. Thus, psychogenic causes were assumed. The patient reported moderate amounts of alcohol consumption and smoked approximately 20 cigarettes a day (23 pack years). Penicillin and contrast agent allergies were known. All other examinations were normal.

The patient had a medical history of heart surgery for septal defect at 6 years of age (6), correction of polydactyly (two additional thumbs, age 6 years), appendectomy (8 years), polypectomy in the nose (10 years) and colon (51 years) were diagnosed and treated.

A 12-lead electrocardiogram (ECG) after admission was normal. Blood laboratory tests showed combined hypercholesterolemia (triglycerides [TG] 81 mg/dl, cholinesterase [CHE] 334 mg/dl, low-density lipoprotein [LDL] 249.89 mg/dl, slightly elevated liver enzymes, gamma-glutamyl transferase [GGT] 103 U/l, glutamic-pyruvic transaminase [GPT] 55 U/l) were not reproducible by further testing. Creatine kinase (CK) was moderately elevated throughout the hospital stay (190–362 U/l), whereby psychogenic muscular tension was considered as the most likely reason. Arterial blood gases (pCO_2_: 51.4%, pO_2_: 50.6%, pH 7.4) in combination with obstructive breathing, severely reduced forced expiratory volume in 1 s (FEV1) (17%) and moderate vascular shadows in chest X‑rays confirmed previously known COPD. Cardiac sonography showed a pronounced septal shift with inspiratory left-sided shift, a borderline right ventricular function and elevated pulmonary artery pressure (36 mm Hg).

Further internal medical consultations recommended gastroscopy whereby an antrum gastritis, duodenitis with several small ulcerous lesions and a reflux esophagitis were diagnosed and treated with pantoprazole 80 mg/day. Further radiological testing, e.g. computed tomography-angiography (CTA) with contrast agent and cortisone treatment (allergy) was performed for suspected abdominal claudication. The examination demonstrated high-grade obstruction of the celiac artery but with good collateral arteries and no signs of reduced perfusion of abdominal organs. Obstruction was confirmed with abdominal duplex sonography, whereby reduced flow profiles in the splenic artery and common hepatic artery were demonstrated. Additionally, in the CT, renal cysts in both kidneys in part hemorrhagic were found. A possible stenosis of the ligamentum arcuatum was evaluated with MR angiography (MRA), but due to phobic reactions and lack of compliance during breathing provocation, two attempts did not yield sufficient data for full evaluation of stenosis of the ligamentum arcuatum. After frequent consultations with abdominal surgeons, gastroenterologists and interventional radiologists, explorative surgery was not performed and a conservative approach was chosen. The abdominal pain significantly decreased after initiating antidepressive and anti-anxiety treatment with sertraline and pregabaline. Treatment of gastritis with adequate proton pump inhibitor dosage may also have contributed to improvement of pain. In a telephone call 3 months after discharge, the patient stated he was pain-free.

### Genetic testing

Due to early onset heart septum defect, polydactyly, slightly reduced cognitive performance, mild microcephaly and facial abnormities as well as psychiatric diagnoses, a geneticist was consulted. A microarray of genomic DNA with SNP Array Cytoscan HD (Affymetrix, Santa Clara, CA, USA) was performed and a 3.2 Mb deletion in region 22q11.21 was detected. This microdeletion was confirmed by molecular cytogenetics (with HIRA 22q11 Kreatech FISH probes, Leica Biosystems, Wetzlar, Germany).

## Discussion

This case history highlights a late diagnosis of 22q11 deletion syndrome (DS). Although early signs such as polydactyly and congenital heart disease were present, genetic testing was only performed after psychiatric manifestation of the disease in middle age. Panic disorder and a severe depressive episode with suicidal ideation were successfully treated with sertraline and antidepressant augmentation with the atypical antipsychotic amisulpride. Antidepressant and anti-anxiety psychopharmacological therapy resembled conventional antidepressant therapy. By combining anxiolytics with a non-sedative anxiolytic (pregabaline), significant improvement of panic disorder was achieved and abdominal pain was also alleviated. If arterial obstruction was a consequence of an abdominal malformation, high blood lipids or smoking, remains open in this case. Furthermore, no certain association between pulmonary obstruction and 22q11 DS is known; however, the patient exhibited nicotine addiction (~23 pack years), which constitutes an additional risk factor for atherosclerosis and could explain COPD-IV. In our patient, typical signs and symptoms were present at an early age. Since the patient did not report significant health problems in this life period, delayed diagnosis might be explainable by the lack of earlier medical consultation.

### DiGeorge syndrome and psychiatry

**Fig. 1:**
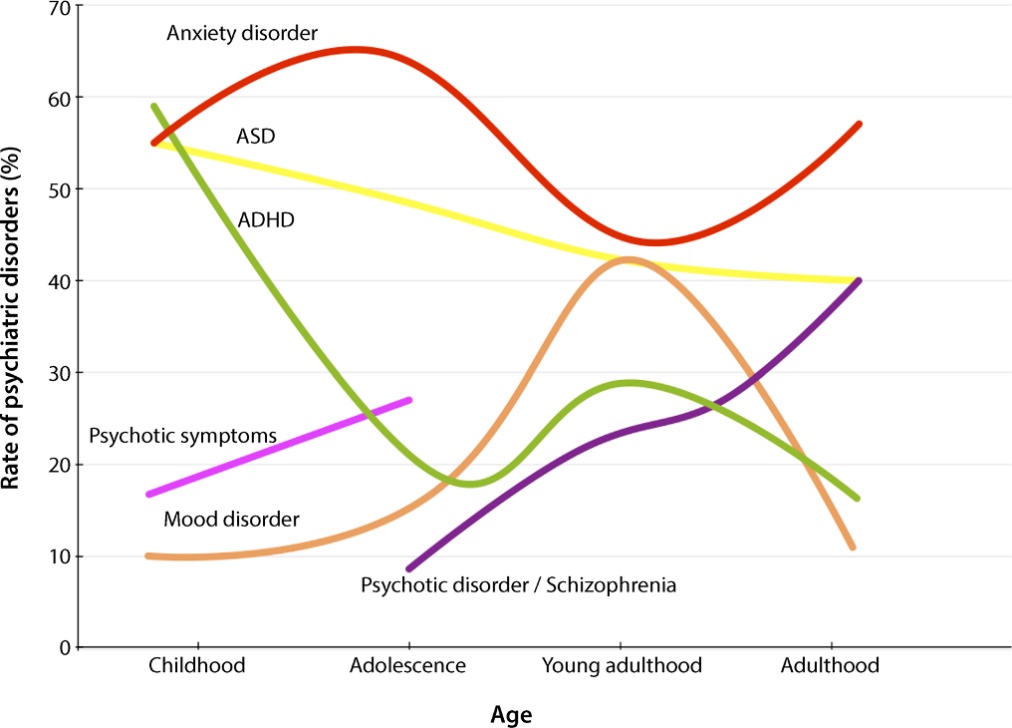
Developmental trajectories of psychiatric diseases among patients with DiGeorge syndrome. *ASD* autism sprectrum disorder, *ADHD* attention-deficit/hyperactivity disorder. (Figure corresponds to Fig. 2B in [1] and is reproduced with permission from Elsevier)

Concerning neuropsychiatric symptoms catechol-O-methyl transferase (*COMT*) deletions result in increased vulnerability for neuropsychiatric disorders. DiGeorge syndrome patients exhibit an approximately 3‑fold increased risk for psychiatric disorders, as shown by a recent study in a large Danish cohort [5]. In this study the mean age at diagnosis for psychiatric disorders was 12.4 years for individuals with deletions. The risk was highly elevated (~20.7 times) for intellectual disabilities, markedly elevated for schizophrenia and psychotic spectrum disorder, and still substantially elevated for mood and anxiety disorders (see Table 1). Notably, numbers tend to vary between epidemiological studies due to design and selection of patients [6]. Incidence rates of psychiatric disorders in DiGeorge syndrome are highlighted in Fig. 1. There are developmental trajectories with anxiety disorders, attention-deficit/hyperactivity disorder (ADHD), autism spectrum disorder peaking in childhood and early youth, while incidence of psychotic disorders increases throughout the lifetime. Strikingly, anxiety disorders exhibit high prevalence rates in DiGeorge syndrome patients, which fits to our patient’s psychiatric history; however, 1–2% of patients diagnosed with schizophrenia have DiGeorge syndrome. Thus, there is consensus that genetic screening is currently not recommended in schizophrenia [7].

Due to deletions of major neurotransmitter gatekeeping enzymes, such as COMT, which degrades dopamine (and to a lesser extent serotonin), neuronal development is impaired in DiGeorge syndrome [1]. Although pathogenetic processes in DiGeorge syndrome are still poorly understood, there is evidence for reductions in neuronal gray matter and alterations of transmitter release [1]. Deletions of important neurotransmitter gatekeeping enzymes provide an excellent model for the discovery of genomic risk factors for neuropsychiatric disorders. The combination of relatively high incidence, developmental disease trajectories, clinical manifestations, environmental stress/life events and animal models could serve as a powerful model for translational research in neuropsychiatric disorders.

There are few specific recommendations for the treatment of DiGeorge syndrome patients [3]. Combinations of psychopharmacological and psychotherapeutic treatments are recommended for the respective psychiatric disorders, but apart from an increased risk for seizures (approximately 5–7-fold compared to healthy subjects) [8], only few specific recommendations are available. Importantly, lifetime concomitant psychosocial treatments are crucial to improve patient outcomes [3].

## Conclusion

This short report highlights the importance of knowledge on this relatively frequent genetic condition. Diagnosing DiGeorge syndrome early in life improves the chances to timely treat psychiatric disorders. On the other hand, a substantial number of adult undiagnosed patients might present with psychiatric symptoms. The neurodevelopment of DiGeorge syndrome is linked to the lack of important neurotransmitter genes such as COMT, which degrades monoaminergic transmitters such as dopamine and serotonin. Moreover, 22q11 DS offers interesting opportunities for translational studies on monoaminergic alterations in neuropsychiatric disorders. Genetic testing for DiGeorge syndrome should be done in psychiatric patients with intellectual disabilities, developmental disorders and schizophrenia if cardiac malformations, immunodeficiency, hypoparathyroidism or malformations are present. Once the diagnosis is confirmed, symptomatic psychopharmacological treatment should be initiated but there should also be a focus on psychosocial treatment to improve patient outcomes.
